# Plasmid-mediated gene transfer of Cas9 induces vector-related but not *Sp*Cas9-related immune responses in human retinal pigment epithelial cells

**DOI:** 10.1038/s41598-022-17269-x

**Published:** 2022-08-01

**Authors:** Julia K. Pfromm, Mario Bonillo, Daniyar Dauletbekov, Kirsten Bucher, M. Dominik Fischer

**Affiliations:** 1 University Eye Hospital, Centre for Ophthalmology, University Hospital Tübingen, Tübingen, Germany; 2 Institute for Ophthalmic Research, Centre for Ophthalmology, University Hospital Tübingen, Elfriede-Aulhorn-Strasse 7, 72076 Tübingen, Germany; 3 Oxford Eye Hospital, Oxford University Hospitals NHS Foundation Trust, Oxford, UK; 4 Nuffield Laboratory of Ophthalmology, Department of Clinical Neurosciences, University of Oxford, Oxford, UK

**Keywords:** Immunology, Diseases, Medical research

## Abstract

The clustered regularly interspaced short palindromic repeat (CRISPR)-associated protein 9 (Cas9) system represents a powerful gene-editing tool and could enable treatment of blinding diseases of the retina. As a peptide of bacterial origin, we investigated the immunogenic potential of Cas9 in models of retinal immunocompetent cells: human microglia (IMhu) and ARPE-19 cells. Transfection with *Streptococcus pyogenes*-Cas9 expression plasmids (*Sp*Cas9 plasmid) induced Cas9 protein expression in both cell lines. However, only ARPE-19 cells, not IMhu cells, responded with pro-inflammatory immune responses as evidenced by the upregulation of IL-8, IL-6, and the cellular activation markers HLA-ABC and CD54 (ICAM). These pro-inflammatory responses were also induced through transfection with equally sized non-coding control plasmids. Moreover, viability rates of ARPE-19 cells were reduced after transfection with both the *Sp*Cas9 plasmids and the control plasmids. Although these results demonstrate cell type-specific responses to the DNA plasmid vector, they show no evidence of an immunogenic effect due to the presence of Cas9 in models of human retinal pigment epithelial and microglia cells. These findings add another layer of confidence in the immunological safety of potential future Cas9-mediated retinal gene therapies.

## Introduction

Inherited retinal dystrophies (IRDs) are a group of rare genetic disorders of the retina that threaten vision and range from causing severe visual disability to the complete loss of light perception. Loss of function mutations can be addressed by supplementing the genetic information with the coding sequence (e.g. *RPE65* in *voretigene neparvovec*) of the disease gene, generally through the use of adeno-associated viral vectors (AAV)^1^. Gain-of-function mutations, such as *RHO* gene mutations causing retinitis pigmentosa (RP), can induce the production of cytotoxic proteins and dominant-negative mutations, like *RP1* variants in RP, and can lead to the expression of mutated proteins that impair the function of the wild type protein. Adding an episomal coding sequence of *RP1 *via AAV-mediated gene therapy would not address the disease mechanism. However, genome editing may be used to disrupt the dominant allele causing the pathogenic mutation^2^.

The clustered regularly interspaced short palindromic repeat (CRISPR)-associated protein 9 (Cas9) system represents an important gene-editing tool for gene therapy. It consists of two components: the Cas9 protein, a bacterial RNA-guided DNA endonuclease which induces double-stranded DNA breaks, and a single guide RNA (sgRNA) that forms a complex with Cas9 and guides it to a specific DNA target sequence^3^. Cas9 can be transferred into a host cell as a preformed complex of Cas9 protein and sgRNA^4,5^, as an mRNA molecule encoding for the Cas9 transgene^4,5^, or via lentiviral delivery of Cas9 mRNA^6^ or AAV-mediated delivery of Cas9 DNA^44^. Additionally, Cas9 gene editing can be achieved through plasmid-mediated gene transfer^7^. Cas9-encoding plasmids can be delivered into the cell via electroporation, microinjection, and non-viral vectors such as nanoparticles and cationic lipids^8^.

CRISPR-Cas9-based retinal gene therapy has already been analyzed in clinical settings (www.clinicaltrials.gov; NCT03872479). DNA plasmid vectors for Cas9 gene transfer have successfully been tested in rodent studies of retinal gene therapy^7,9,10^. However, it was shown that the transfer of DNA plasmids can up-regulate pro-inflammatory cytokines^11,12^ induce inflammatory cell infiltration^11^, and trigger cell death in various cell types^11,12^. Moreover, CRISPR-Cas9-based gene therapies still contain unquantified risks, as Cas9 can induce innate and adaptive immune response in blood cells^13–17^. This is of notable relevance, as immunity to Cas9 can lead to elimination of Cas9-expressing cells^17^. However, the potential immunogenicity of Cas9 in retinal cells remains to be determined.

Immunocompetent cells in the retina that could potentially react to Cas9 and/or the DNA of the plasmid vector include microglia and retinal pigment epithelium (RPE) cells. Microglia express major innate pattern recognition receptors (PRRs)^18^. Upon stimulation, they are capable of producing various inflammatory cytokines and upregulating activation molecules including major histocompatibility complex (MHC) class II molecules and intercellular adhesion molecule-1 (ICAM-1; CD54)^18^. Additionally, microglia play important roles in the defense against infectious diseases^18^ and become activated and proliferate in response to retinal gene therapy^19^. They initiate retinal inflammation^20^ and essentially control the infiltration of immune cells into the retina^20,21^. Upon stimulation they are activated within minutes^22^ and induce early inflammatory responses that precede responses of macroglia^23^ including Mueller cells^24^. Collectively, this suggests that microglia might act as rapid immunological sensors and key inducers of initial innate immune responses in the retina, making them an interesting target in the study of initial innate immune responses to Cas9. Similar to microglia, RPE cells have also been shown to upregulate MHC class II molecules and ICAM-1 after stimulation^25^. Due to their expression of major PRRs and their responsiveness to stimulation of these receptors^26,27^ RPE cells are considered key players in the first-line innate immune response to microbial organisms^27^. Moreover, the RPE is involved in the pathogenesis of several IRDs^28^. Accordingly, RPE cells not only represent an attractive gene therapy target for Cas9 gene therapy, but might also show immunological responses to Cas9. Immune responses of retinal microglia and RPE cells to the DNA plasmid vector or to Cas9 DNA, RNA, and/or the transgene protein could potentially affect the Cas9 gene-editing efficacy and impair cell viability. In this study, we analyzed whether plasmid-mediated gene transfer of Cas9 induces immune responses in models of human RPE and microglia cells using ARPE-19 cells and the new immortalized human microglia cell line SV40 (IMhu).

## Results

### Characterization of the immune-responsiveness of IMhu cells to stimulation with PRR ligands

It has been shown that inherited retinal diseases caused by gene mutations can be corrected via plasmid-mediated Cas9 gene editing^7,10^. However, there is evidence that either Cas9 or DNA plasmids can induce immune responses^4,11,14^. Retinal microglia participate in inflammatory processes by secreting pro-inflammatory cytokines such as tumor necrosis factor-α (TNF-α), interleukin (IL)-1β, IL-6, and IL-18^18^. Moreover, murine microglia have been shown to release pro-inflammatory cytokines like TNF-α, IL-1β, IL-6 and the type I interferons (IFN) IFN-α and IFN-β in response to ligands of major PRRs^29^. This suggests that microglia could potentially also respond to the DNA plasmid vector and/or Cas9.

**Figure 1:**
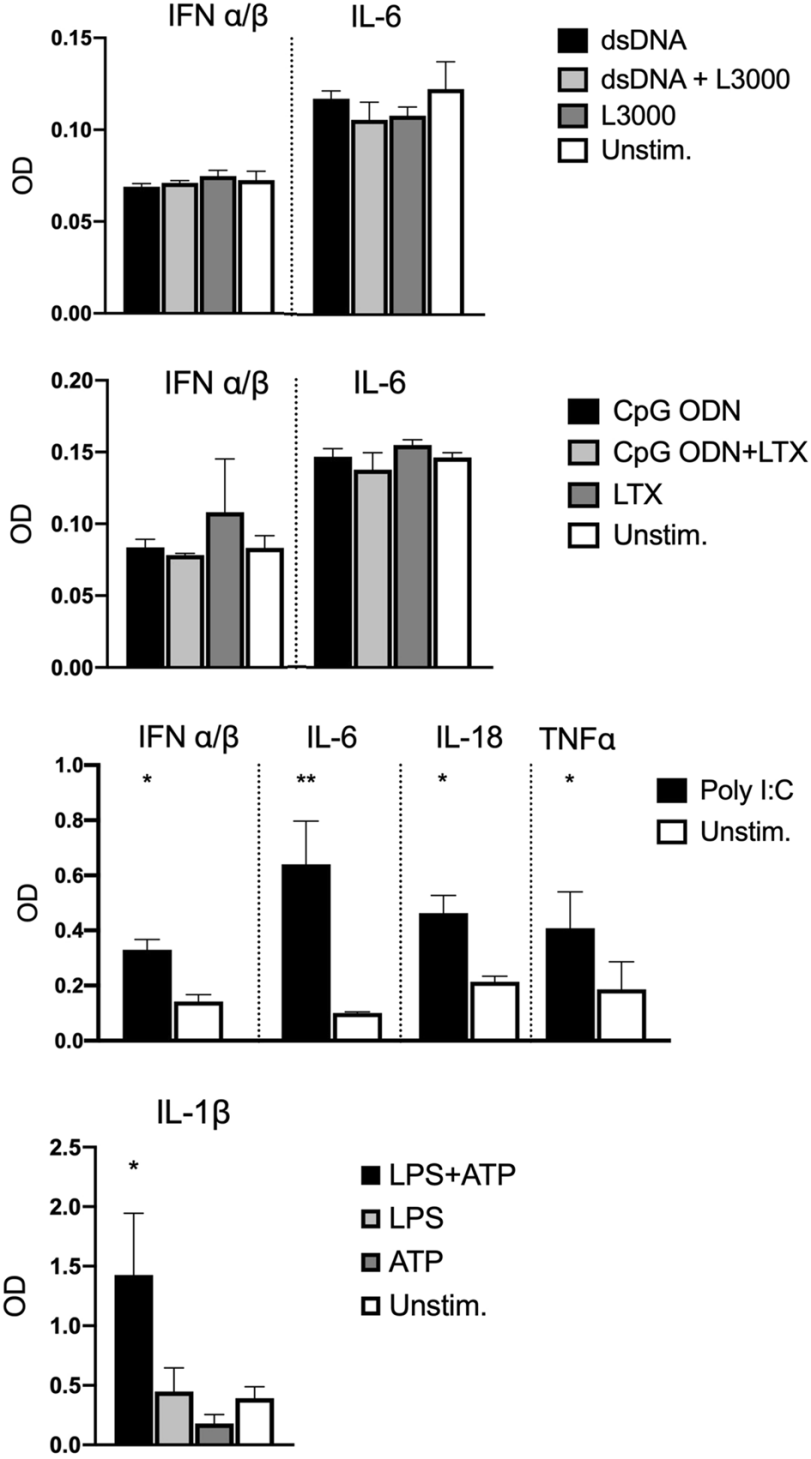
IMhu cytokine response to stimulation of major PRRs. Concentrations of TNF-α, IFN-α/β, IL-1β, IL-6, and IL-18 in the supernatant of IMhu cells 24 h following stimulation with 5 μg/ml dsDNA and 1.5 μl/ml L3000; 1 µM CpG ODN and 5 µl/ml LTX; 10 μg/ml Poly I:C; or 5 μg/ml LPS and 5 mM ATP. Cytokine concentrations were measured using HEK-Blue IFN α/β, IL-6, TFNα, IL-1β, and IL-18 reporter cells. Error bars represent means + SD of n = 3 per group. Normally distributed data was assessed using One-way ANOVA with post hoc Tukey’s tests. Non-normally distributed data was assessed using non-parametric Mann–Whitney tests or Kruskal–Wallis tests with post hoc assessment using Dunn’s tests with control for joint ranks and Bonferroni adjustment. *p < 0.05, **p < 0.01. Asterisks indicate significant differences in comparison to unstimulated cells.

IMhu cells were used to analyze microglial immune responses to plasmid-mediated gene transfer of Cas9. This new microglia cell line is of validated human origin, as confirmed by sequencing and displays numerous similarities to primary human microglia in terms of morphology, the expression of cell surface markers, and immune responses to stimulation with pro-inflammatory cytokines^30,31^. IMhu cells exhibit the same typical microglial phenotype observed in primary and immortalized microglial cultures^31^. IMhu express surface markers specific for human microglia-macrophage lineage such as CD11b, TGFβR, and P2RY12; these markers are also expressed on primary microglia^30–32^. IMhu also demonstrate phagocytic and migratory activity characteristic of primary microglia^31,33^. Additionally, it has been demonstrated that IMhu respond to pro-inflammatory stimulation with the activation of an M1 phenotype, involving upregulation of several pro-inflammatory cytokines and chemokines, a response also observed in primary human microglia^30,34^. The characterization of IMhu as a microglial cell model, though, has not yet been extended to an assessment of the cell line’s responsiveness to the activation of major PRRs. Thus, before investigating the IMhu immune response to *Sp*Cas9 plasmid transfection, we tested the immune-competence of this cell line by stimulating it with ligands of various PRRs. After 24 h, levels of pro-inflammatory cytokines and type I IFNs in the supernatant were determined using HEK-Blue IFN-α/β, HEK-Blue IL-1β, HEK-Blue IL-6, HEK-Blue IL-18, and HEK-Blue TNFα reporter cells (Fig. 1). Stimulation of intracellular DNA receptors with double-stranded DNA (dsDNA) or oligodeoxynucleotides containing unmethylated cytosine-guanine dinucleotides (CpG ODNs) did not induce a detectable production of IFN-α/β or pro-inflammatory IL-6. In contrast, stimulation with the Toll-like receptor (TLR)3 ligand Poly I:C induced significant releases of IFN-α/β, IL-6, IL-18, and TNF-α. Moreover, significantly elevated levels of IL-1β showed that IMhu also responded to inducers of inflammasome signaling (LPS and ATP) (Fig. 1). This suggests that IMhu cells are capable of mounting inflammatory immune responses to ligands of TLR3 and inducers of inflammasome signaling, but have limited immunoreactivity to ligands of intracellular DNA receptors.

### Transfection with the SpCas9 plasmid results in Cas9 protein expression in IMhu cells but does not trigger cytokine responses

**Figure 2:**
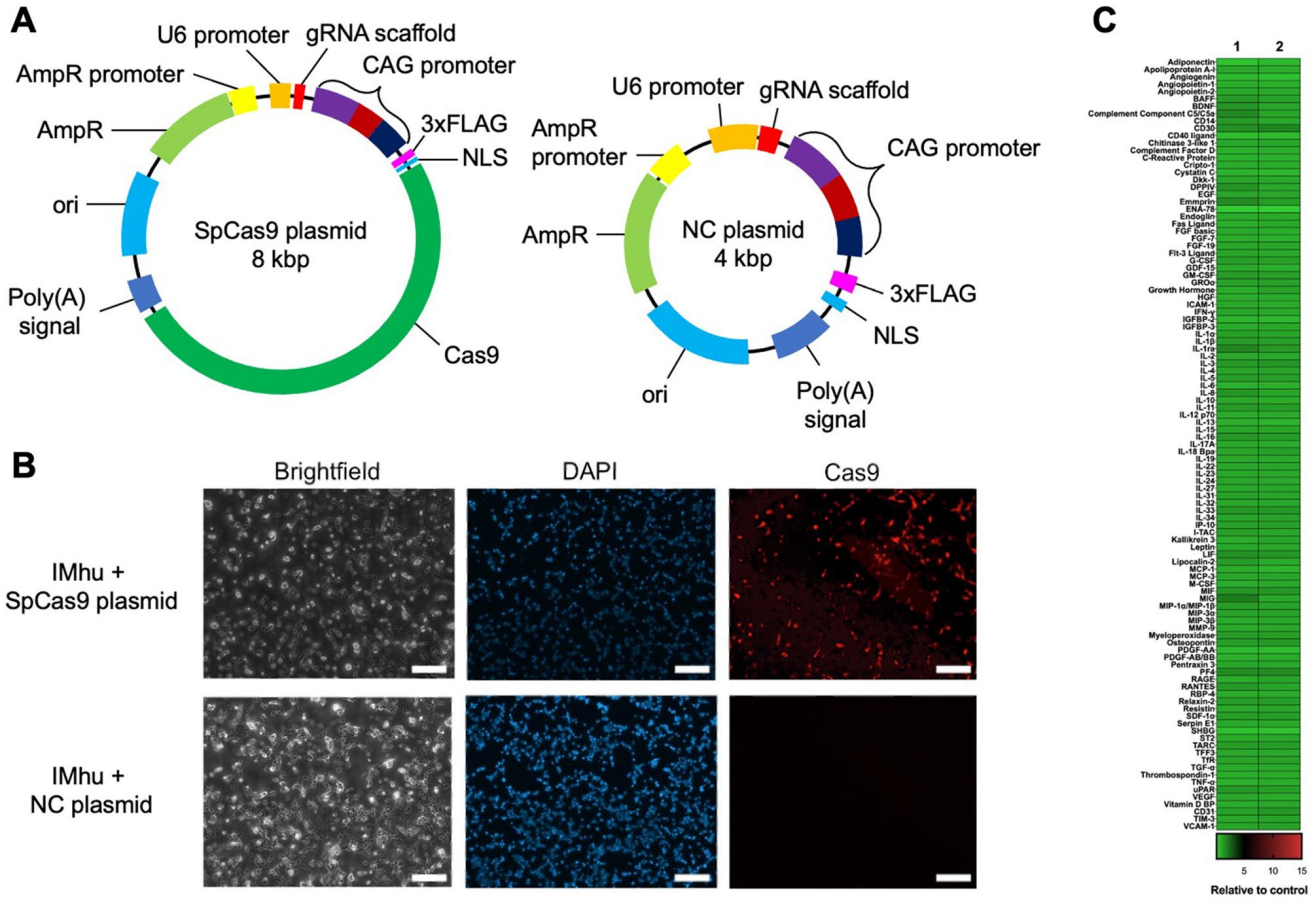
*Sp*Cas9 plasmid transfection of IMhu cells results in Cas9 expression but does not induce cytokine responses. (**A**) Experimental Streptococcus pyogenes (*Sp*) Cas9 expression plasmid (*Sp*Cas9 plasmid) and non-coding control plasmid (NC plasmid) used to assess the effect of Cas9 on IMhu cells. In the NC plasmid the Cas9 encoding sequence is removed. (**B**) Immunostaining of Cas9 in transfected IMhu cells. Cells were separately transfected with either the *Sp*Cas9 plasmid or the NC plasmid. At 24 h post-transfection, cells were stained with Cas9 antibodies (red) and DAPI (nuclei, blue). *Sp*Cas9 plasmid-transfected IMhu cells expressed Cas9 after *Sp*Cas9 plasmid transfection, whereas NC plasmid-transfected cells did not express Cas9. Data are representative of three independent experiments. Scale bar 100 µm. *NLS* nuclear localization sequence, *ori* origin of replication, *AmpR* ampicillin resistance gene, *FLAG* protein tag. (**C**) Heatmap of cytokine levels in IMhu supernatant 24 h after transfection with the *Sp*Cas9 plasmid. Cytokine levels were determined using Proteome Profiler Antibody Arrays. Colors represent cytokine ratios of *Sp*Cas9 plasmid-transfected versus L3000 treated cells (left column) or NC plasmid-transfected versus L3000 treated cells (right column) respectively.

To test whether plasmid-mediated gene transfer of Cas9 induces immune responses in cell models of human microglia cells, we designed an experimental plasmid coding for the *Streptococcus pyogenes* Cas9 (*Sp*Cas9) sequence (*Sp*Cas9 plasmid) (Fig. 2A). An identical non-coding plasmid (NC plasmid) without the Cas9 sequence served as negative control for Cas9 staining (Fig. 2A). To confirm that *Sp*Cas9 plasmid transfection results in Cas9 expression, IMhu cells were transfected with either the *Sp*Cas9 plasmid or NC plasmid using cationic lipid mediated transfection. At 24 h post-transfection, cells were stained with an anti-*Sp*Cas9 antibody and analyzed via fluorescence microscopy (Fig. 2B). We found that approximately 30% of the IMhu cells expressed Cas9 intracellularly. As expected, no Cas9 expression was detected in IMhu cells transfected with the NC plasmid (Fig. 2B).

Next, we analyzed the immune responses of IMhu cells to *Sp*Cas9 plasmid transfection. To this end, IMhu cells were transfected via cationic lipid mediated transfection with either the *Sp*Cas9 plasmid or the NC plasmid, or treated with the transfection reagent Lipofectamine 3000 (L3000) alone. At 24 h post-transfection supernatant was harvested and the release of 105 cytokines was assessed using Proteome Profiler™ Antibody Arrays. Heat map analysis of cytokine ratios of *Sp*Cas9 plasmid-transfected *versus* L3000 treated cells, or NC plasmid-transfected *versus* L3000 treated cells respectively showed that neither transfection with the *Sp*Cas9 plasmid nor with the NC plasmid triggered a detectable change in the release of any of the measured immune mediators (Fig. 2C). To verify these results, we repeated this experiment including IMhu cells which were either treated only with the *Sp*Cas9 plasmid (but no L3000) or not stimulated, as additional controls. At 6 h, 12 h, 24 h, and 48 h after stimulation, cytokine levels of IL-1β, IL-6, IL-18, and TNF-α were determined using HEK Blue™ cells. No differences in the levels of any of these cytokines were observed between *Sp*Cas9 plasmid- or NC plasmid-transfected cells, or cells treated only with either the *Sp*Cas9 plasmid or L3000 compared to unstimulated controls (Figure S1A). To evaluate whether IMhu cells responded to other plasmids of similar or larger size encoding for *Sp*Cas9 and/or different fluorescent proteins, IMhu cells were transfected with two additional plasmids (mKate: 4.8 kbp and the EGFP *Sp*Cas9 plasmid: 9.3 kbp) (Figure S1B). Again, measurements of cytokine production from supernatant samples using HEK Blue™ IFN α/β-, IL-1β-, IL-6-, IL-18-, or TFNα-cells revealed no significant changes in cytokine releases of the plasmid-stimulated cells compared to the non-stimulated controls (Figure S1C).

These results indicate that neither plasmid transfection in general, nor plasmid-mediated gene transfer of Cas9 triggers immune responses in IMhu microglia cells.

### SpCas9 plasmid transfection induces a vector-related release of IL-6 and IL-8 in ARPE-19 cells

**Figure 3:**
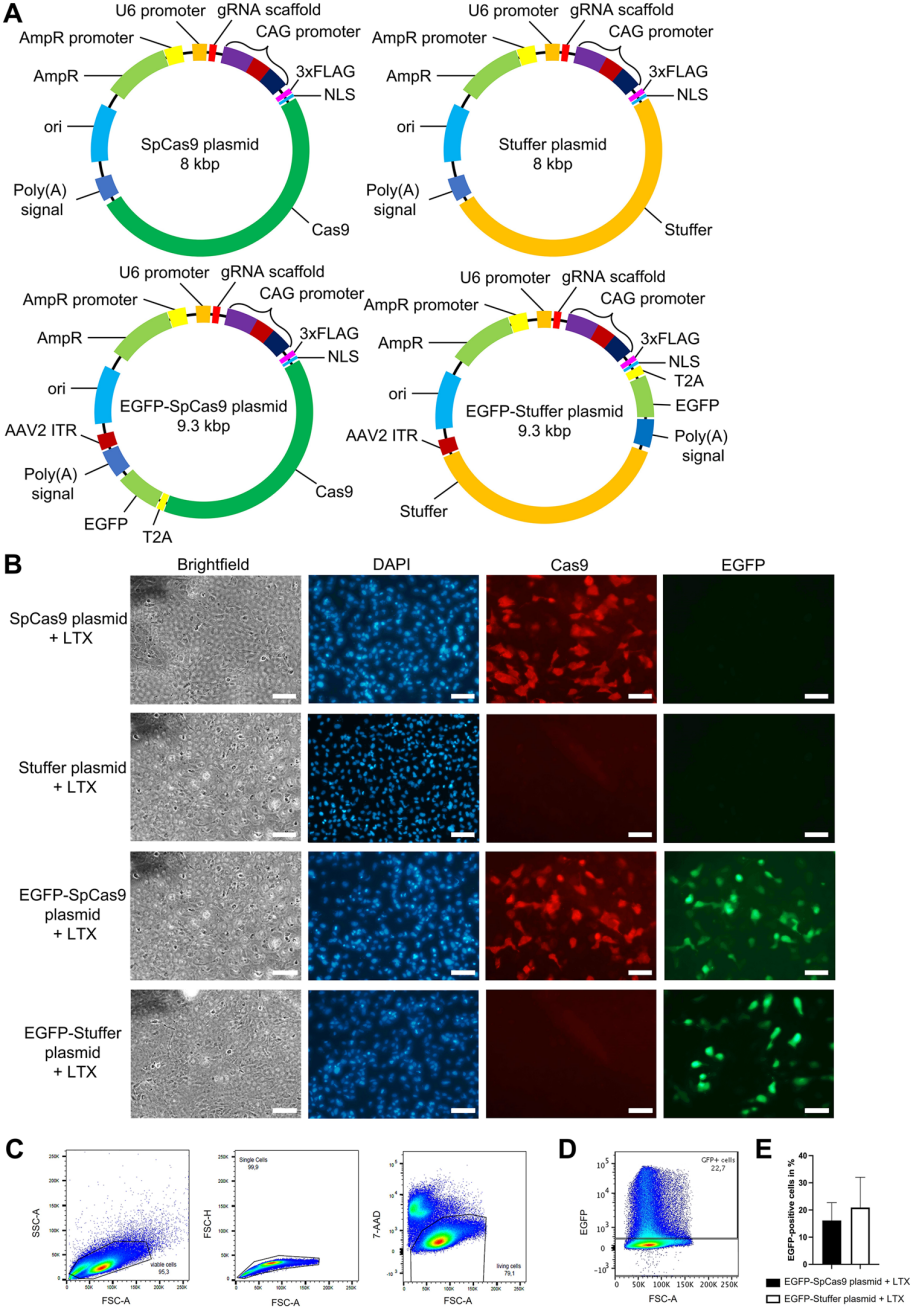
*Sp*Cas9-, EGFP-*Sp*Cas9-, and EGFP-stuffer-plasmid transfection results in Cas9 and/or EGFP expression in ARPE-19 cells. (**A**) *Sp*Cas9 plasmid, stuffer plasmid, EGFP-*Sp*Cas9 plasmid, and EGFP-stuffer plasmid used to evaluate the effect of Cas9 on ARPE-19 cells. In the stuffer plasmid and the EGFP-stuffer plasmid the Cas9 encoding sequence is replaced by an equally sized non-coding stuffer sequence. *NLS* nuclear localization sequence, *ori* origin of replication, *AmpR* ampicillin resistance gene, *FLAG* protein tag. (**B**) Microscopic evaluation of Cas9 and EGFP expression in transfected ARPE-19 cells. Cells were separately transfected with either the *Sp*Cas9 plasmid, stuffer plasmid, EGFP-*Sp*Cas9 plasmid, or the EGFP-stuffer plasmid. After 24 h cells were stained with Cas9 antibodies (red) and DAPI (blue). Data are representative of three independent experiments. Scale bar 100 µm. (**C**–**E**) Flow cytometric evaluation of the transfection rates of EGFP-*Sp*Cas9 plasmid or the EGFP-stuffer plasmid-transfected cells. (**C**,**D**) ARPE-19 cells were gated as single, 7-AAD negative living cells (**C**) and analyzed for the expression of EGFP (**D**). (**E**) Percentages of EGFP positive ARPE-19 cells. Bars represent means + SD of mean values pooled from three independent experiments measured in duplicates.

To evaluate whether plasmid-mediated gene transfer of Cas9 induces immune responses in human RPE cells, ARPE-19 cells were transfected with the *Sp*Cas9 plasmid and a non-coding control plasmid. As transfection efficacy^35^ and nuclear delivery of plasmids^36,37^ have been shown vary in relation to vector size^35–37^, the Cas9 sequence was replaced by a non-coding stuffer sequence enlarging the plasmid to the size of the 8 kbp *Sp*Cas9 plasmid (stuffer plasmid) (Fig. 3A, upper panel) to create the control plasmid. Two additional EGFP-encoding plasmids: an EGFP-*Sp*Cas9 plasmid (9.3 kbp) expressing Cas9 and EGFP under the same promoter and a respective equally sized non-coding EGFP-stuffer plasmid were used (Fig. 3A, lower panel) to facilitate precise flow cytometric comparison of transfection rates. To assess Cas9 and/or EGFP protein expression following plasmid transfection, ARPE-19 cells were transfected with equal concentrations of the *Sp*Cas9 plasmid, the stuffer plasmid, the EGFP-*Sp*Cas9 plasmid, or the EGFP-stuffer plasmid. As the *Sp*Cas9 plasmids and their respective stuffer control plasmids were of equal size and almost identical molar mass, the transfection at equal mass corresponded to an almost equimolar transfection of the *Sp*Cas9 plasmids and their respective control plasmids. After 24 h cells were stained with *Sp*Cas9 antibodies as described. Microscopic evaluation confirmed intracellular Cas9 expression exclusively in *Sp*Cas9 plasmid and EGFP-*Sp*Cas9 plasmid-transfected cells and a comparable EGFP expression exclusively in EGFP-*Sp*Cas9 plasmid and EGFP-stuffer plasmid-transfected cells (Fig. 3B) and demonstrated Cas9 and EGFP co-expression following transfection with the EGFP-*Sp*Cas9 plasmid as expected (Fig. 3B, third panel). For flow cytometric quantification of the transfection rates, EGFP-*Sp*Cas9 plasmid or the EGFP-stuffer plasmid-transfected cells were harvested at 24 h post treatment, stained with the cell death marker 7-AAD, and subsequently analyzed. Living ARPE-19 cells were gated and the percentage of EGFP positive cells was determined as shown in Fig. 3C,D. This analysis confirmed comparable transfection rates of the EGFP-*Sp*Cas9 plasmid [16.13 ± 3.8% (mean ± SD)] and the EGFP-stuffer plasmid (20.92 ± 6.43%) (Fig. 3E) with no significant differences seen between groups (p = 0.557).

**Figure 4:**
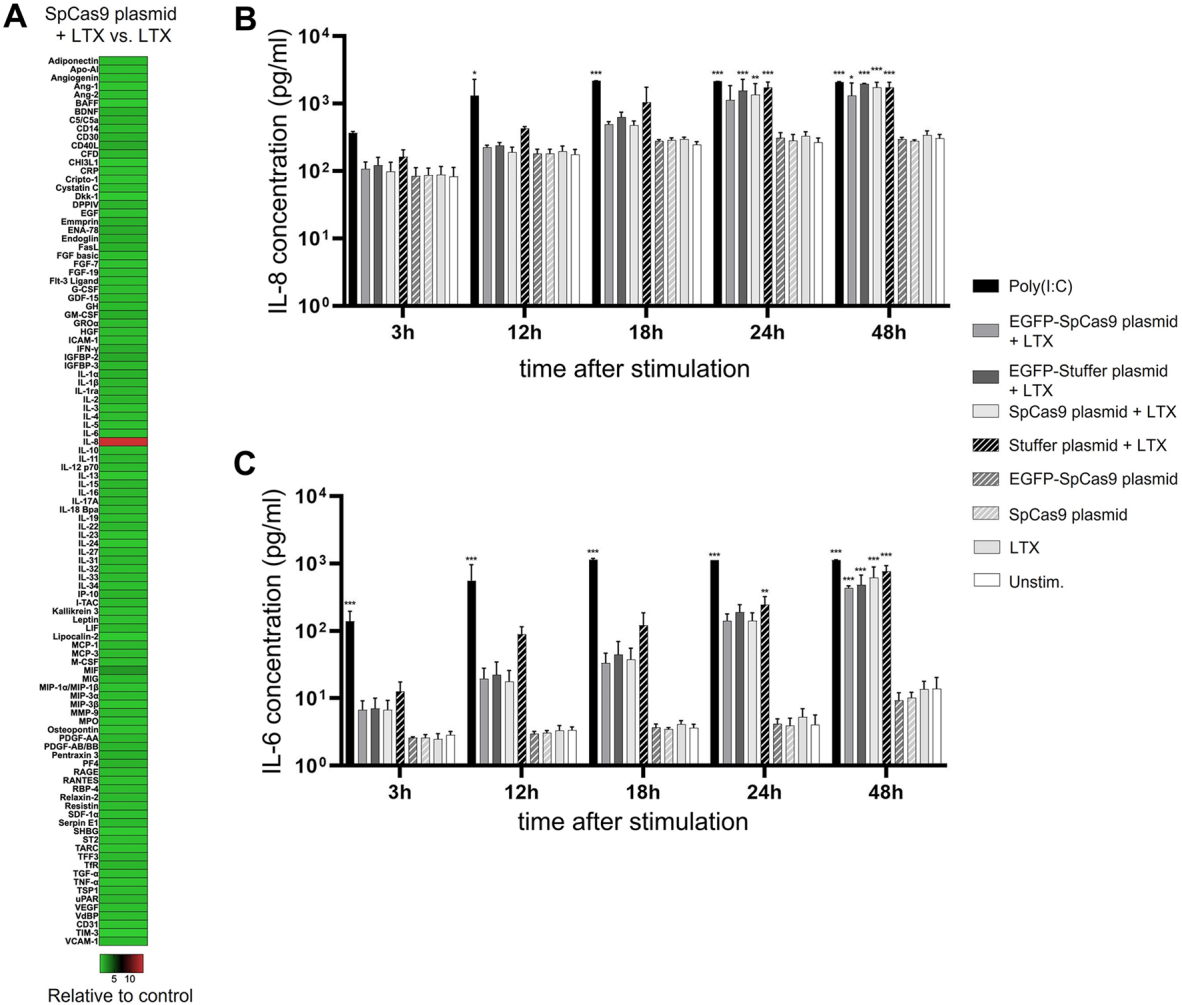
Plasmid-mediated gene transfer of *Sp*Cas9 induces vector-related IL-6 and IL-8 release in ARPE-19 cells. (**A**) Heatmap of cytokine levels in the supernatant of ARPE-19 cells 24 h after transfection with the *Sp*Cas9 plasmid as measured by Proteome Profiler Antibody Arrays. Colors show cytokine ratios of *Sp*Cas9 plasmid-transfected versus LTX treated cells. (**B**,**C**) ELISA analysis of IL-8 and IL-6 concentrations in the supernatant of ARPE-19 cells transfected with the *Sp*Cas9 plasmid, the stuffer plasmid, the EGFP-*Sp*Cas9 plasmid, and the EGFP-stuffer plasmid, or treated with the SpCas9 plasmid or LTX alone or left unstimulated. Poly(I:C) (10 µg/ml) served as a positive stimulation control. (**B**) Transfection with both the *Sp*Cas9-encoding plasmids and the stuffer control plasmids induced a significant IL-8 release. (**C**) Transfection with both the *Sp*Cas9-encoding plasmids and the stuffer control plasmids also triggered a significant production of IL-6. Data was analyzed via one-way ANOVA followed by Bonferroni’s comparison tests for selected pairs of columns. Asterisks indicate significant differences in comparison to unstimulated ARPE-19 cells. Bars represent means + SD of pooled mean values from three independent experiments measured in duplicates. *p < 0.05, **p < 0.01, ***p < 0.001.

To analyze immune responses of ARPE-19 cells to plasmid-mediated gene transfer of Cas9, ARPE-19 cells were transfected with either the *Sp*Cas9 plasmid or treated with the transfection reagent Lipofectamine LTX (LTX) alone. At 24 h following transfection, cytokine levels in the supernatant were determined using Proteome Profiler™ Antibody Arrays. Heat map analysis demonstrates that transfection of ARPE-19 cells with the SpCas9 plasmid triggered an IL-8 response (Fig. 4A). IL-8 was increased 14-fold following *Sp*Cas9 plasmid-transfection when compared to LTX treatment only.

To strengthen the results of this semiquantitative analysis with a quantitative method, IL-8 concentrations in the supernatant of ARPE-19 cells transfected with the *Sp*Cas9 plasmid or the EGFP-*Sp*Cas9 plasmid were measured at five time points after treatment (3 h, 12 h, 18 h, 24 h, and 48 h) using a sandwich ELISA. To determine whether the observed IL-8 response was Cas9-related or plasmid-vector-related, this experiment also included ARPE-19 cells transfected with the stuffer plasmid and the EGFP-stuffer plasmid. ARPE-19 cells which were either treated with only the *Sp*Cas9 plasmid, with LTX, or left unstimulated served as additional controls. Transfection with both *Sp*Cas9-encoding plasmids as well as with both stuffer plasmids induced a strong IL-8 release, significant for all plasmid-transfected groups at 48 h after stimulation (48 h: SpCas9 plasmid + LTX vs unstim. control p < 0.001; stuffer plasmid + LTX vs unstim. control p < 0.001; EGFP-SpCas9 plasmid vs unstim. control p = 0.014; EGFP-stuffer-plasmid vs unstim. control p < 0.001), whereas no significant differences in IL-8 levels were observed between the additional controls (Fig. 4B). Comparison between plasmid-transfected ARPE-19 cells revealed no significant differences in the IL-8 response between cells treated with *Sp*Cas9 encoding plasmids and stuffer plasmids (*Sp*Cas9 plasmid *vs* stuffer plasmid, p = 0.368; EGFP-*Sp*Cas9 plasmid vs EGFP-stuffer-plasmid, p = 0.354) (Fig. 4B).

Chen et al. have shown that IL-8 secretion by ARPE-19 cells is mediated by the induction of NF-κB and MAPK signaling, and activation of these signaling pathways leads to the additional release of IL-6^38^. To test whether plasmid-mediated gene transfer of Cas9 in ARPE-19 cells triggers IL-6 secretion, we measured IL-6 in the supernatant of *Sp*Cas9 plasmid-transfected cells and the corresponding control groups using a sandwich ELISA. This more sensitive cytokine analysis showed that transfection with both *Sp*Cas9-encoding plasmids as well as with both stuffer plasmids resulted in an increase in IL-6 production which was significant for all groups at 48 h after stimulation (48 h: *Sp*Cas9 plasmid + LTX vs unstim. control p < 0.001; stuffer plasmid + LTX vs unstim. control p < 0.001; EGFP-*Sp*Cas9 plasmid *vs* unstim. control p < 0.001; EGFP-stuffer-plasmid vs unstim. control p < 0.001) (Fig. 4C). No differences in IL-6 concentrations were observed between cells treated only with either the *Sp*Cas9 plasmid or LTX and unstimulated controls. Moreover, there were no significant differences in the IL-6 response between cells treated with *Sp*Cas9 encoding plasmids and stuffer plasmids (*Sp*Cas9 plasmid *vs* stuffer plasmid, p = 0.424; EGFP-*Sp*Cas9 plasmid *vs* EGFP-stuffer-plasmid, p = 0.755) (Fig. 4C).

Taken together, plasmid transfection of ARPE-19 cells with either the *Sp*Cas9-encoding plasmids or the stuffer control plasmids triggered significant IL-8 and IL-6 secretion. These effects were comparable between plasmids both coding and non-coding for Cas9, suggesting that in ARPE-19 cells, the immunogenic effect of Cas9 transfection is triggered by the transfected plasmid DNA rather than by the presence or expression of the Cas9 transgene.

### Plasmid transfection upregulates immunological surface markers on ARPE-19 cells

**Figure 5:**
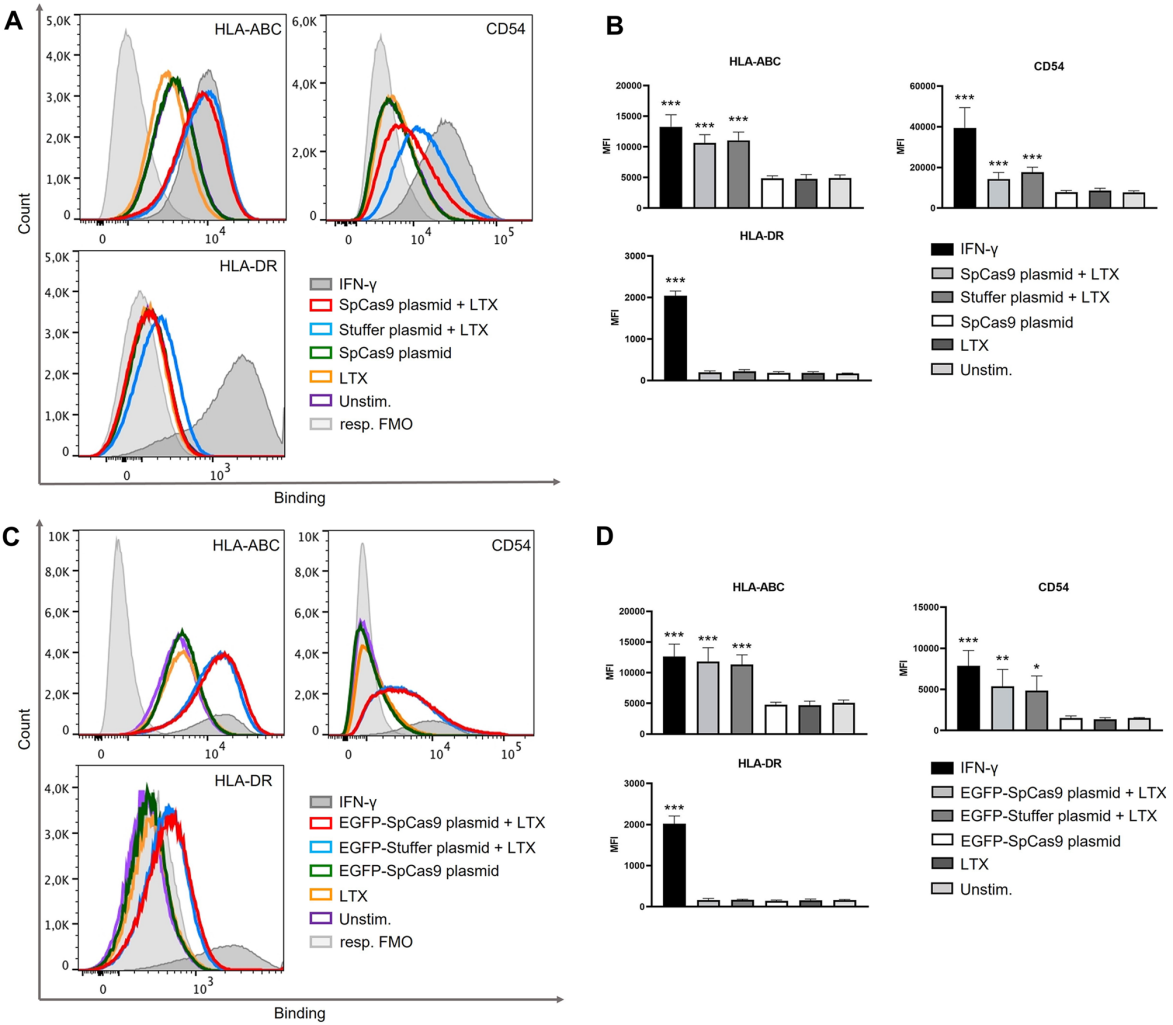
Plasmid-mediated gene transfer of Cas9 induces HLA-ABC and CD54 upregulation in ARPE-19 cells. ARPE 19 cells were transfected with the *Sp*Cas9-encoding plasmids or the corresponding stuffer plasmids, or treated with the *SpCas9* plasmid or LTX alone or left unstimulated. IFN-γ (1000 IU/ml) served as a positive stimulation control. At 24 h after transfection cells were analyzed by flow cytometry. Living ARPE-19 cells were gated as shown in Fig. 3C and analyzed for the expression of the surface markers HLA-ABC, HLA-DR and CD54. Surface marker expression following transfection with the *Sp*Cas9 plasmid or the stuffer plasmid (**A**,**B**) and the EGFP-*Sp*Cas9 plasmid or the EGFP-stuffer plasmid (**C**,**D**). (**A**,**C**) Surface marker expression presented as overlays of single-color histograms of log10 mean fluorescence intensity (MFI) obtained with fluorescence minus one (FMO) control (filled histograms) and specific antibodies against respective surface marker (open histograms). (**B**,**D**) Graphical analysis of the surface marker expression showing increases of HLA-ABC and CD54 after transfection with the *Sp*Cas9-encoding plasmids or the corresponding stuffer plasmids. Bars represent means + SD of pooled mean values from three independent experiments measured in duplicates. Data was analyzed by one-way ANOVA followed by Bonferroni’s comparison tests. Asterisks indicate differences in comparison to unstimulated ARPE-19 cells. Kruskal–Wallis-test followed by pairwise comparisons was used to analyze non-normally distributed data. *p < 0.05, **p < 0.01, ***p < 0.001.

It has been shown that stimulated ARPE-19 cells upregulate immunological surface markers indicative of cell activation, including HLA-ABC and HLA-DR major histocompatibility (MHC) antigens, and CD54 (ICAM-1)^25^. To analyze whether plasmid-mediated gene transfer of Cas9 leads to cell activation, ARPE-19 cells were stimulated with the *Sp*Cas9 plasmid and the stuffer plasmid. At 24 h post-stimulation, cells were harvested, stained with fluorescent antibodies against HLA-ABC, HLA-DR and CD54, and the cell death marker 7-AAD, and subsequently analyzed via flow cytometry. Living ARPE-19 cells were gated as shown in Fig. 3C and surface expression of HLA-DR, HLA-ABC, and CD54 was determined (Fig. 5A). We found that neither *Sp*Cas9 plasmid- and stuffer plasmid-transfection nor stimulation of ARPE-19 cells with the Cas9 plasmid or LTX alone induced changes in the expression of HLA-DR (Fig. 5A,B; lower panels). However, both *Sp*Cas9 plasmid transfection and stuffer plasmid transfection led to a significant upregulation of HLA-ABC (*Sp*Cas9 plasmid + LTX vs unstim. control p < 0.001; stuffer plasmid + LTX vs unstim. control p < 0.001) and CD54 (SpCas9 plasmid + LTX vs unstim. control p < 0.001; stuffer plasmid + LTX vs unstim. control p < 0.001), with no significant differences in the expression levels of these markers seen between the two groups. No changes in HLA-ABC and CD54 expression were observed in cells treated only with either the *Sp*Cas9 plasmid or LTX (Fig. 5A,B; upper panels). Similar results were observed following transfection with the EGFP-*Sp*Cas9 plasmid and the EGFP-stuffer-plasmid: here, transfection also did not lead to changes in the expression of HLA-DR (Fig. 5C,D; lower panels), but caused increases in the expression of HLA-ABC and CD54. These were significant for HLA-ABC (EGFP-*Sp*Cas9 plasmid + LTX vs unstim. control p < 0.001; EGFP-stuffer plasmid + LTX vs unstim. control p < 0.001). A similar trend was observed for CD54 (EGFP-*Sp*Cas9 plasmid + LTX vs unstim. control p = 0.008; EGFP-stuffer plasmid + LTX vs unstim. control p = 0.011) (Fig. 5A,B; upper panels).

Overall, this suggests that the upregulation of HLA-ABC and CD54 expression on ARPE-19 cells was triggered by the transfection of the DNA plasmid rather than by the presence or expression of the Cas9 transgene.

### Plasmid transfection decreases the viability rate of ARPE-19 cells

**Figure 6:**
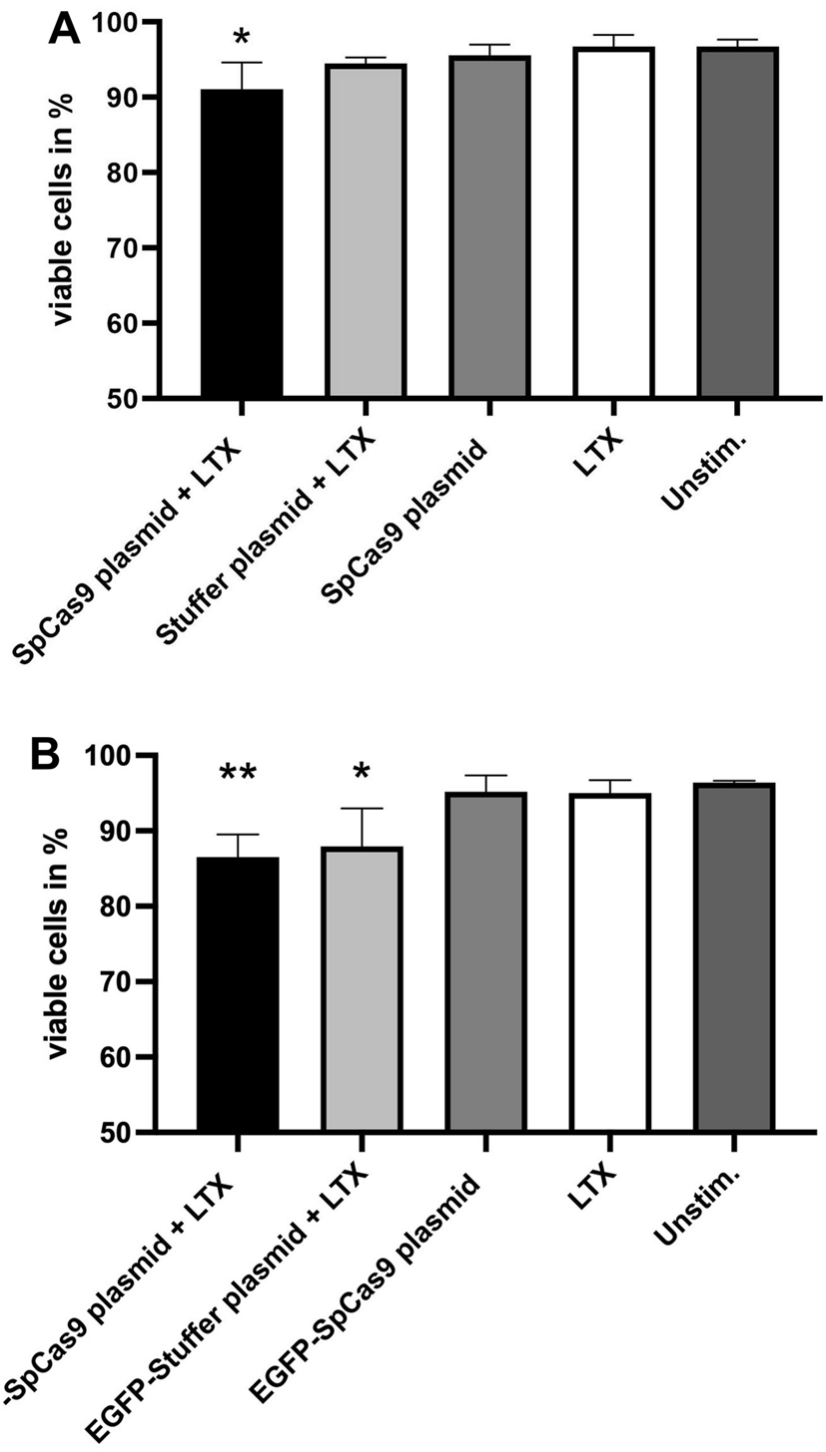
Viability rates of ARPE-19 cells after plasmid transfection. Viability rates were analyzed via flow cytometry 24 h after plasmid transfection. Percentages of viable ARPE-19 cells following transfection with the *Sp*Cas9 plasmid or the stuffer plasmid (**A**) and the EGFP-*Sp*Cas9 plasmid or the EGFP-stuffer plasmid (**B**). ARPE-19 cells receiving LTX or *Sp*Cas9 plasmid treatment only and unstimulated ARPE-19 cells served as control groups. Bars represent means + SD of pooled mean values from three independent experiments measured in duplicates. Normally distributed data was analyzed by one-way ANOVA followed by Bonferroni’s comparison tests for selected pairs of columns. Kruskal–Wallis-test followed by pairwise comparisons was used to analyze non-normally distributed data. Asterisks indicate differences in comparison to unstimulated ARPE-19 cells. *p < 0.05 **p < 0.01.

It has been shown that cationic lipid transfection of DNA plasmids can induce cell death^12^. To determine whether cationic lipid-mediated transfection of the *Sp*Cas9 plasmid influences cell viability, the transfected IMhu cells and ARPE-19 cells were first examined microscopically. There was no evidence of increased cell death in *Sp*Cas9 plasmid-transfected and NC plasmid-transfected IMhu microglia up to 48 h after treatment (Figure S2A). In contrast numerous floating spherical cells in the supernatant of ARPE-19 cultures suggested reduced cell viability from 24 h on after transfection with the *Sp*Cas9-encoding plasmids or the corresponding stuffer plasmids (Figure S2B). To investigate the viability of transfected ARPE-19 cells in more detail, these cells were either transfected with the *Sp*Cas9 plasmid or stuffer plasmid, or treated with LTX or *Sp*Cas9 plasmid only and compared to unstimulated cells. After 24 h, 7-AAD negative living ARPE-19 cells were quantified by flow cytometry (compare Fig. 3C). The analysis showed a minimal reduction in the viability percentages of *Sp*Cas9 plasmid-transfected and stuffer plasmid-transfected ARPE-19 cells when compared to control groups (*Sp*Cas9 plasmid + LTX vs unstim. control p = 0.049; stuffer plasmid + LTX vs unstim. control p = 0.05) (Fig. 6A). Similar results were observed for EGFP-*Sp*Cas9 plasmid-transfected and EGFP-stuffer plasmid-transfected cells (EGFP-*Sp*Cas9 plasmid + LTX vs unstim. control p = 0.009; EGFP-stuffer plasmid + LTX vs unstim. control p = 0.02) (Fig. 6B). The percentage of living cells did not differ between *Sp*Cas9 encoding plasmids or the corresponding stuffer plasmids, suggesting weak cytotoxic effects induced by plasmid transfection rather than the presence or expression of the Cas9.

## Discussion

CRISPR/Cas9 technology has been successfully tested in animal studies of retinal gene therapy^39,40^ and a human trial of CRISPR/Cas9 retinal gene editing is currently ongoing (www.clinicaltrials.gov; NCT03872479). Plasmids containing Cas9 or other transgenes have been used for gene transfer in a number of translational efforts to develop genetic therapies for inherited retinal dystrophies (IRD)^7,9,10,41,42^. Moreover, plasmid-mediated gene transfer is applied in the development of cell-based retinal therapies^43,44^, where target cells, such as RPE cells, are transfected ex vivo with the respective plasmid vector prior to subsequent transplantation into the retina. Interestingly, the transfer of DNA plasmids can trigger strong innate immune responses in muscle cells^11^, demonstrating the potential immunogenicity of plasmid DNA. Moreover, Cas9 is a peptide of bacterial origin and therefore carries the potential for non-self-recognition and immunogenicity. Such an effect could lead to adverse reactions and negate therapeutic efficacy in the target tissue. Indeed, recent publications demonstrate that Cas9 can induce humoral and cell-specific immune responses in human blood cells^13–17^. On the other hand, no Cas9-specific immune responses were observed in a pre-clinical study of CRISPR/Cas9 retinal gene editing^45^, suggesting a potential site- and cell-specific reaction to Cas9. We therefore investigated the innate immune response to Cas9 in model systems relevant to ocular gene therapy. Specifically, we evaluated immune responses to plasmid-transfection of *Sp*Cas9 in human retinal cell models and demonstrated cell type-specific immunogenicity of the DNA plasmid vector, but not of *Sp*Cas9, in a model of human RPE cells and a complete absence of plasmid-related or *Sp*Cas9-related immunogenicity in a human microglia model.

In plasmid-mediated gene transfer of Cas9, potential immunogenic components that could trigger innate immune responses consist of plasmid DNA, the transgene mRNA, and the transgene protein. Additionally, immune responses observed following transfection of experimental plasmids lacking the Cas9 sequence might be induced by the expression of vector encoded fluorescence proteins such as GFP^46^. It has been shown that transfection of non-coding plasmid DNA into muscle cells induced upregulation of the endosomally expressed DNA sensor TLR9 and of other PRRs responding to cytoplasmic DNA and triggered pro-inflammatory cell infiltration^11^. Moreover, electrotransfer of *Sp*Cas9 transgene RNA stimulated an immune response in human CD34+ hematopoietic stem cells^4^ and extracellular application of *Sp*Cas9 proteins induced a pro-inflammatory cytokine release in human monocytes^14^. Human RPE cells have been shown to express intracellular receptors for DNA including TLR9 and the cytosolic DNA receptor cGAS^27,47^, and also exhibit PRRs capable of detecting RNA^26,48^ and extracellular proteins^48,49^. Moreover, ARPE-19 cells were found to react to the TLR9 ligand CpG DNA and to intracellular mitochondrial DNA^27,50^. Additionally, human primary RPE cells responded to ligands of the RNA sensing TLR3^48^. Similarly, human microglia cells have been shown to express cGAS^51^ and innate PRRs responding to RNA and proteins^52^, but in contrast to RPE cells, the expression of TLR9 is low or absent in primary human microglia^52^. In line with this we observed that although IMhu microglia cells reacted to ligands of a RNA sensing TLR (TLR3), they did not respond to the TLR9 ligand CpG ODN. Additionally, we also found that they did not react to dsDNA. Collectively, this suggests that differences may exist in the capability of microglia and RPE cells to sense intracellular DNA.

Differences in the immune responsiveness to DNA provide a possible explanation for the discrepancy in the reactivity of IMhu cells and ARPE-19 cells to *Sp*Cas9 plasmid transfection. While *Sp*Cas9 plasmid transfection did not elicit a cytokine response in IMhu cells, in ARPE-19 cells it caused a release of pro-inflammatory IL-8 and IL-6 and an upregulation of the cellular activation markers HLA-ABC and CD54 (ICAM-1) in response to the *Sp*Cas9 plasmid as well as to the EGFP-*Sp*Cas9 plasmid. Similar responses were also induced in cells transfected with the corresponding stuffer control plasmids. In the latter groups, the observed immune reaction could neither be attributed to the presence of Cas9 mRNA nor to Cas9 protein. Moreover, no responses were detected in ARPE-19 cells when the Cas9 plasmid was applied extracellularly. Taken together, this suggests that intracellular plasmid DNA vectors triggered inflammatory immune responses in ARPE-19 cells. In contrast, in IMhu cells, the absence of an immune response to plasmid transfection might have been related to their reduced reactivity to DNA antigens and/or intracellularly expressed proteins.

A detailed analysis of the immune responses to Cas9 plasmid transfection in ARPE-19 cells revealed no significant differences in the IL-8 and IL-6 responses between cells treated with *Sp*Cas9 encoding plasmids and stuffer plasmids. There were also no significant differences seen in the expression of HLA-ABC and CD54 between ARPE-19 cells transfected with *Sp*Cas9 encoding plasmids and stuffer plasmids. Collectively, this indicates that the inflammatory immune responses were triggered by the intracellular plasmid DNA vector but not by the presence or the expression of the *Sp*Cas9 transgene.

The cytokines and surface molecules upregulated in ARPE-19 cells following plasmid transfection have been shown to play important roles in ocular inflammation. Pro-inflammatory IL-6 is a critical mediator of uveitis^53^ and induces the disruption of tight junction complexes between RPE cells, leads to the VEGF-induced recruitment of retinal microglia to the RPE layer and thereby compromises the barrier function of the RPE^54^. IL-8 is also associated with human retinal inflammatory diseases^55–57^ and has a chemotactic effect on neutrophil granulocytes^58^ as well as highly cytotoxic CD8+  T cells^59^. Moreover, ICAM-1, which is also expressed by human primary RPE cells, is critically involved in the cross-migration of leukocytes across the blood-retinal barrier^60^, while antigen presentation via HLA-ABC molecules is required for the activation of CD8+ cytotoxic T cells. This suggests that an upregulation of these immune molecules in the retina induced by cell or gene therapies might promote ocular inflammatory processes.

We observed that cationic lipid transfection of Cas9 plasmids not only induced pro-inflammatory immune responses in ARPE-19 cells but also resulted in a minor decrease in cell viability. Interestingly, cell viability was also reduced in ARPE-19 cells transfected with the stuffer control plasmids, but not in cells that were only treated with either transfection reagent or plasmid DNA. This suggests that increased cell death was not caused by the transfection reagent alone or by extracellular plasmids or the Cas9 transgene. Increased cell death in cells transfected with DNA-cationic lipid complexes, but not in cells treated with uncomplexed material, was also observed by Nguyen et al.^12^ In this study the effect of cationic lipid transfection of bacterial plasmids was studied in human HeLa cells. A detailed analysis revealed that cell death following transfection with plasmid DNA-cationic lipid complexes was caused by apoptosis as shown by substantial DNA fragmentation and upregulation of genes involved in the ER stress-mediated apoptosis pathway^12^. Thus, it is possible that the minor decrease in cell viability seen in our plasmid-transfected ARPE-19 cells was caused by apoptosis induced by plasmid DNA-cationic lipid complexes. Overall, this suggests that in vivo approaches of plasmid-mediated retinal gene transfer should be monitored for the occurrence of inflammatory immune responses and cell death.

In summary, we have shown that plasmid-mediated gene transfer of *Sp*Cas9 induced an increased release of pro-inflammatory cytokines and an upregulation of cellular activation markers in a model of human RPE cells, but not in human microglia. Importantly, this immune response was only induced by plasmid transfection but not specifically by Cas9. As the immune reactivity of retinal cells has been shown to depend on their tissue context^61^, immune responses to retinal gene therapy might also depend on the ocular or cellular microenvironment of the transfected cells, indicating that in vitro immune responses may differ from in vivo responses. Nevertheless, our results strongly suggest a generally low immunogenicity of Cas9 in microglia and RPE cells. Results demonstrating no or low immunogenicity of Cas9 are also seen in vivo studies of CRISPR/Cas9 retinal gene editing. Thus, no peripheral adaptive immune responses to *Sa*Cas9 were detected in non-human primates subretinally injected with AAV5-encoded CRISPR-SaCas9 to correct the common deep-intronic mutation in CEP290 associated with LCA10 (EDIT-101; Editas Medicine, Inc.)^45^. Additionally, initial clinical data from the ongoing, open label Phase 1/2 BRILLIANCE clinical trial of EDIT-101 (www.clinicaltrials.gov; NCT03872479) also demonstrate the absence of peripheral Cas9-specific antibody or T-cell responses in the treated patients^62^. However, detailed analyses of the immune response of retinal cells to Cas9 were still lacking. Now, our findings, showing for the first time that Cas9 does not elicit immune responses in models of RPE and microglia cells, provide further evidence of the low/absent immunogenicity of Cas9 in and are therefore encouraging for future studies of Cas9-mediated retinal gene therapies.

## Materials and methods

### Plasmids

The EGFP-*Sp*Cas9 plasmid was obtained from Addgene (pSpCas9(BB)-2A-GFP plasmid, #48138, Addgene, Watertown, MA, USA). To construct the *Sp*Cas9 plasmid, the EGFP-*Sp*Cas9 plasmid was used as template DNA for PCR amplification of the *Sp*Cas9 and CAG promoter gene sequences. PCR amplification was performed using the primers: 5′-CCACGCGTGACGGCCTATTTCCCATGATTC-3′ and 5′-GGAATTCGGCAGTGGTCCGGACC-3′. The PCR product was digested with restriction enzymes MluI-HF (New England Biolabs, Ipswich, MA, USA) and Nsil-HF (New England Biolabs, Ipswich, MA, USA) and inserted into the backbone of the *cj*Cas9 plasmid (#89752, Addgene, Watertown, MA, USA). XL10-Gold^®^ ultracompetent cells (Agilent, Santa Clara, CA, USA) were used in subsequent bacterial transformation. Plasmid isolation was performed with the EndoFree^®^ Plasmid Mega Kit (Qiagen, Hilden, DE) as per the manufacturer’s protocol. The *Sp*Cas9 plasmid was sequenced using the Mix2Seq Kit (Eurofins, Luxemburg, LU), successfully confirming the presence of the expected plasmid sequence. Plasmid DNA concentration and purity were assessed using the Infinite M200 microplate reader (Tecan, Männedorf, CH) according to the manufacturer’s protocol. To design a corresponding non-expression Cas9 plasmid (NC plasmid), the *Sp*Cas9 plasmid, excepting the *Sp*Cas9 sequence, was amplified using the KOD hot start DNA polymerase (Merck, Darmstadt, DE) with the primers: 5′-GTCCGGAAAAAGGCCGGCGGCCAC-3′ and 5′- GTCCGGAGCTGGGACTCCGTGGAT-3′. The PCR fragment was subsequently digested using the restriction enzyme BspEI (New England Biolabs, Ipswich, MA, USA) and was self-ligated using the T4 DNA ligase (New England Biolabs, Ipswich, MA, USA). Bacterial transformation, plasmid isolation, and sequencing were performed as describe above. To create size-matched control plasmids for the *Sp*Cas9 plasmid (stuffer plasmid) and EGFP-*Sp*Cas9 plasmid (EGFP-stuffer plasmid), the *Sp*Cas9 sequence of both plasmids was replaced by a stuffer sequence of the identical size. The generation of the stuffer plasmid and EGFP-stuffer plasmid was performed by VectorBuilder (Shenandoah, TX, USA).

The mKate fluorescent plasmid (4.8 kbp) was obtained from Addgene (#54826).

### Cell culture

ARPE-19 cells (ATCC, Manassas, VA, USA) were cultured in DMEM Glutamax containing 10% FBS and 1% penicillin/streptomycin (all Thermo Fisher Scientific, Waltham, MA, USA). Immortalized Human Microglia SV40 (IMhu) (abm, Richmmond, BC, Canada) were cultured in flasks treated with 6–10 μg/cm^2^ Human Collagen type 1 in DMEM high glucose medium supplemented with 10% FBS and 1% penicillin/streptomycin (all Thermo Fisher Scientific, Waltham, MA, USA). HEK-Blue™ IFN α/β, IL-6, TFNα, IL-1β, and IL-18 cells (InvivoGen, San Diego, CA, USA) were cultured in DMEM high glucose growth medium containing 1% penicillin/streptomycin, 30 μg/ml Blasticidin, 100 μg/ml Zeocin, and 100 μg/ml Normocin (all InvivoGen, San Diego, CA, USA). All cell cultures were maintained at 37 °C and 5% CO_2_. Cell cultures were confirmed to be mycoplasma free through the use of the MycoSEQ mycoplasma detection kit (Thermo Fisher Scientific, Waltham, MA, USA).

### Stimulation of IMhu with PRR ligands

IMhu were seeded in culture medium 24 h prior to stimulation. To stimulate intracellular DNA receptors, or TLR3, or TLR7, the medium was replaced with OptiMEM reduced serum medium (Thermo Fisher Scientific, Waltham, MA, USA) containing either 7.5 µl/ml Lipofectamine 3000, 8.35 µl/ml Plus Reagent (both Thermo Fisher Scientific, Waltham, MA, USA), and 1 μg/ml dsDNA (InvivoGen, San Diego, CA, USA), or 5 µl/ml Lipofectamine LTX, 3.5 µl/ml Plus reagent (both Thermo Fisher Scientific, Waltham, MA, USA) and 1 µM CpG ODN 2216 (InvivoGen, San Diego, CA, USA), or 10 μg/ml Poly (I:C) (InvivoGen, San Diego, CA, USA), or 5 μg/ml Imiquimod (InvivoGen, San Diego, CA, USA) respectively. To stimulate the inflammasome, cells were incubated with 5 μg/ml LPS O55:B5 (Sigma Aldrich, St Louis, MO, USA) in OptiMEM for 6 h, before removal of the LPS medium and replacement with 5 mM ATP (InvivoGen, San Diego, CA, USA) in OptiMEM. Cells were incubated at 37 °C and supernatant was collected after 24 h.

### Plasmid transfection of IMhu and ARPE-19

Cationic lipid mediated transfection of IMhu cells with *Sp*Cas9 plasmids and NC plasmids and of ARPE-19 cells with EGFP-*Sp*Cas9 plasmids, EGFP-stuffer plasmids, *Sp*Cas9 plasmids and stuffer plasmids was performed using Lipofectamine 3000 or Lipofectamine LTX with Plus reagent respectively (both Thermo Fisher Scientific, Waltham, MA, USA). IMhu cells were seeded at 2 × 10^5^ cells per well of a 24-well plate. ARPE-19 cells were seeded at 9 × 10^5^ cells per well of a 24-well plate and at 2.5 × 10^4^ cells per well of a 96-well plate, respectively. Both cell types were incubated in cell culture medium overnight prior to transfection with Cas9 or control plasmids at optimized plasmid-reagent ratios. Additionally, IMhu were transfected with an mKate fluorescent plasmid and the EGFP-*Sp*Cas9 plasmid. IMhu were transfected with 1.5 μl/ml Lipofectamine 3000, 1 μl/ml Plus reagent and 1.5 μg/ml plasmid DNA. ARPE-19 cells were transfected with 1.5 µl/ml Lipofectamine LTX, 0.375 µl/ml Plus reagent and 375 ng/ml plasmid DNA. Stimulation with 10 µg/ml Poly (I:C) served as positive control. Cells were incubated for 24 h at 37 °C prior to collection of supernatant and fixation of cells for immunohistochemistry staining. To assess the time point after plasmid transfection at which the Cas9 protein started to be expressed by IMhu cells and ARPE-19 cells, the cells were transfected with the EGFP-*Sp*Cas9 plasmid and EGFP fluorescence was analyzed using fluorescence microscopy.

### Measurement of cytokines

Semiquantitative immune-detection of human cytokines, chemokines, growth factors, and angiogenesis markers in the supernatant of IMhu and ARPE-19 cells was performed using the Proteome Profiler Human XL Cytokine Array Kit (R&D Systems, Minneapolis, MN, USA). A near-infrared fluorescence signal corresponding to the amount of cytokine bound was generated utilizing IRDye 800CW followed by LI-COR detection. A LI-COR Odyssey^®^ Infrared Imaging System (LI-COR, Bad Homburg, Germany) was used to detect near-infrared fluorescence. The Odyssey scan was run with a resolution of 84 μm, an intensity of 5, 800 nm, an absorbance of 774 nm, and an emission of 789 nm. Signal intensity, measured in pixel density, was analyzed using ImageStudioLite Software (LI-COR Biosciences, Lincoln, NE, USA). The average signal of the duplicate spots was measured for each analyte and determined and normalized to the average signal of the reference spots after correction with the background signal.

Concentrations of IL-8 and IL-6 in the supernatants of ARPE-19 cells were determined by sandwich ELISA using the Human IL-8 DuoSet ELISA and the Human IL-6 DuoSet ELISA (both R&D systems, Minneapolis, MN, USA) according to the manufacturer’s protocol. Cytokine concentrations in the supernatants of IMhu cells were measured using HEK-Blue™ IFN α/β, IL-6, TFNα, IL-1β, and IL-18 reporter cells (all InvivoGen, San Diego, CA, USA). These cells allow the detection of the respective cytokine through the activation of an NF-κB-inducible promoter and the production of secreted embryonic alkaline phosphatase (SEAP). Concentrations of SEAP in the supernatant can be assessed by a SEAP detection assay (InvivoGen, San Diego, CA, USA) using QUANTI-Blue™, a reagent that turns blue in the presence of SEAP. HEK-Blue™ cell measurement of cytokines was performed as per manufacturer’s protocol. In brief, HEK-Blue™ cells were incubated with supernatant from stimulation experiments at 37 °C for 24 h. Induced HEK-Blue™ supernatant was then collected and incubated with QUANTI-Blue™ at 37 °C and SEAP concentrations were assessed using Infinite M200 microplate reader (Tecan, Männedorf, CH) at 640 nm.

### Immunohistochemistry

IMhu and ARPE-19 cells were seeded onto 18 mm diameter round coverslips (Fisher Scientific, Waltham, MA, USA) and transfected as described above. Then, cells were washed twice in a wash buffer (WB) of 1% NDS (abcam, Cambridge, UK) and 0.05% Tween20 (Sigma Aldrich, St Louis, MO, USA) in PBS (Thermo Fisher Scientific, Waltham, MA, USA) and fixed in 4% formaldehyde (Sigma Aldrich, St Louis, MO, USA) in PBS for 10 min. Following fixing, cells were washed three times with WB and permealized for ten minutes using 0.05% Triton X-100 (Thermo Fisher Scientific, Waltham, MA, USA) in PBS. Following three washes with WB, the cells were blocked with 10% NDS and 0.05% Tween20 in PBS for one hour. The cells were then washed twice in WB before overnight incubation at 8 °C in the presence of the primary antibody, Cas9 (7A9-3A3) Mouse mAb (Cell Signaling Technology, Danvers, MA, USA), at a concentration of 1:600. After three washes in WB, the cells were incubated with the secondary antibody, Donkey Anti-Mouse IgG (Alexa Fluor 568) (abcam, Cambridge, UK) at 1:500 in Dako antibody diluent (Agilent, Santa Clara, CA, USA) for two hours. Cells were washed three times with WB prior to incubation with DAPI (Sigma Aldrich, St Louis, MO, USA) in WB for 2 min in the dark. Coverslips were mounted using Fluor Save Reagent (Merk Millipore, Burlington, MA, USA). Staining was assessed and images were captured using fluorescence microscopy.

### Flow cytometry

For flow cytometric characterization of cells, the following antibodies, peptides or reagents were used: 7-aminoactinomycin D (7-AAD), Anti-HLA-DR, APC-Cy™7 (1:1; AB_2868692, clone L243), APC Mouse Anti-Human HLA-ABC (1:100; AB_398603, clone G46-2.6), PE Mouse Anti-Human CD54 (1:100; AB_395901, clone HA58), Human BD Fc Block™ (1:40; AB_2869554) and Anti-Mouse Ig, κ/Negative Control Compensation Particles Set (all BD Biosciences, Heidelberg, Germany). As positive control, cells were stimulated with 1000 IU/ml IFN-γ (Bio-Techne, Wiesbaden, Germany). Flow cytometry measurements were performed on a FACSCanto™ II (BD Biosciences) and data was evaluated using FlowJo software.

### Statistical analysis

JMP version 14.2 (SAS Institute) and SPSS version 25.0 (SPSS Inc. Chicago) were used for statistical analysis. Data was initially assessed for normality using the Shapiro–Wilk test and normally distributed data was further assessed using Student’s *t*-tests or One-way ANOVA. Post hoc analysis of One-way ANOVAs was performed using Tukey’s tests or Bonferroni adjustment. Non-normally distributed data was assessed using Kruskal–Wallis tests or non-parametric Mann–Whitney tests. Post hoc assessment of Data analyzed with Kruskal–Wallis tests was performed using pairwise comparisons or Dunn’s test with control for joint ranks and Bonferroni adjustment. GraphPad PRISM version 8 (GraphPad Software Inc.) was used to create graphs.

## Supplementary Information

Supplementary Information.

## Data Availability

The datasets generated and analyzed during the current study are available from the corresponding author on reasonable request. The plasmid sequences generated in the present study can be accessed at the NCBI databank, GenBank, under the following accession numbers: ON886908 (SpCas9-plasmid); ON886909 (NC-plasmid); ON886911 (EGFP-Stuffer plasmid); ON886910 (Stuffer plasmid).

## References

[1] Bucher K, Rodríguez-Bocanegra E, Dauletbekov D, Fischer MD (2021). Immune responses to retinal gene therapy using adeno-associated viral vectors—implications for treatment success and safety. Prog. Retin. Eye Res..

[2] Diakatou M, Manes G, Bocquet B, Meunier I, Kalatzis V (2019). Genome editing as a treatment for the most prevalent causative genes of autosomal dominant retinitis pigmentosa. Int. J. Mol. Sci..

[3] Peddle CF, Maclaren RE (2017). The application of CRISPR/CAS9 for the treatment of retinal diseases. Yale J. Biol. Med..

[4] Cromer MK (2018). Global transcriptional response to CRISPR/Cas9-AAV6-based genome editing in CD34+ hematopoietic stem and progenitor cells. Mol. Ther..

[5] Hendel A (2015). Chemically modified guide RNAs enhance CRISPR-Cas genome editing in human primary cells. Nat. Biotechnol..

[6] Ling S (2021). Lentiviral delivery of co-packaged Cas9 mRNA and a Vegfa-targeting guide RNA prevents wet age-related macular degeneration in mice. Nat. Biomed. Eng..

[7] Bakondi B (2016). In vivo CRISPR/Cas9 gene editing corrects retinal dystrophy in the S334ter-3 rat model of autosomal dominant retinitis pigmentosa. Mol. Ther..

[8] Lino CA, Harper JC, Carney JP, Timlin JA (2018). Delivering CRISPR: A review of the challenges and approaches. Drug Deliv..

[9] Cai Y (2019). In vivo genome editing rescues photoreceptor degeneration via a Cas9/RecA-mediated homology-directed repair pathway. Sci. Adv..

[10] Vagni P (2019). Gene editing preserves visual functions in a mouse model of retinal degeneration. Front. Neurosci..

[11] Mann CJ (2012). Molecular signature of the immune and tissue response to non-coding plasmid DNA in skeletal muscle after electrotransfer. Gene Ther..

[12] Nguyen LT, Atobe K, Barichello JM, Ishida T, Kiwada H (2007). Complex formation with plasmid DNA increases the cytotoxicity of cationic liposomes. Biol. Pharm. Bull..

[13] Charlesworth CT (2019). Identification of preexisting adaptive immunity to Cas9 proteins in humans. Nat. Med..

[14] Kang R, Zhu S, Zeh H, Tang D (2018). The STING-STAT6 pathway drives Cas9-induced host response in human monocytes. Biochem. Biophys. Res. Commun..

[15] Moreno AM (2019). Immune-orthogonal orthologues of AAV capsids and of Cas9 circumvent the immune response to the administration of gene therapy. Nat. Biomed. Eng..

[16] Simhadri VL (2018). Prevalence of pre-existing antibodies to CRISPR-associated nuclease Cas9 in the USA population. Mol. Ther. Methods Clin. Dev..

[17] Wagner DL (2019). High prevalence of *Streptococcus pyogenes* Cas9-reactive T cells within the adult human population. Nat. Med..

[18] Rathnasamy G, Fould WS, Ling E, Kaur C (2018). Retinal microglia—a key player in healthy and diseased retina. Prog. Neurobiol..

[19] Reichel FF (2017). AAV8 can induce innate and adaptive immune response in the primate eye. Mol. Ther..

[20] Okunuki Y (2019). Retinal microglia initiate neuroinflammation in ocular autoimmunity. Proc. Natl. Acad. Sci..

[21] Okunuki Y (2018). Microglia inhibit photoreceptor cell death and regulate immune cell infiltration in response to retinal detachment. Proc. Natl. Acad. Sci. USA.

[22] Nimmerjahn A, Kirchhoff F, Helmchen F (2005). Resting microglial cells are highly dynamic surveillants of brain parenchyma in vivo. Neuroforum.

[23] Wang M, Wong WT (2014). Microglia-Müller cell interactions in the retina. Adv. Exp. Med. Biol..

[24] Wang M, Ma W, Zhao L, Fariss RN, Wong WT (2011). Adaptive Müller cell responses to microglial activation mediate neuroprotection and coordinate inflammation in the retina. J. Neuroinflammation.

[25] Kanuga N (2002). Characterization of genetically modified human retinal pigment epithelial cells developed for in vitro and transplantation studies. Investig. Ophthalmol. Vis. Sci..

[26] Wörnle M (2011). Inhibition of TLR3-mediated proinflammatory effects by alkylphosphocholines in human retinal pigment epithelial cells. Investig. Ophthalmol. Vis. Sci..

[27] Ebihara N (2007). Distinct functions between toll-like receptors 3 and 9 in retinal pigment epithelial cells. Ophthalm. Res..

[28] von Lintig J, Kiser PD, Golczak M, Palczewski K (2010). The biochemical and structural basis for trans-to-cis isomerization of retinoids in the chemistry of vision. Trends Biochem. Sci..

[29] Olson JK, Miller SD (2004). Microglia initiate central nervous system innate and adaptive immune responses through multiple TLRs. J. Immunol..

[30] Chiavari M, Ciotti GMP, Navarra PLL (2019). Pro-inflammatory activation of a new immortalized human microglia cell line. Brain Sci..

[31] Garcia-Mesa Y (2017). Immortalization of primary microglia: A new platform to study HIV regulation in the central nervous system. J. Neurovirol..

[32] Butovsky O (2013). Identification of a unique TGF-b-dependent molecular and functional signature in microglia. Nat. Neurosci..

[33] Rawat P, Spector SA (2016). Development and characterization of a human microglia cell model of HIV-1 infection. J. Neurovirol..

[34] Orihuela R, McPherson CA, Harry GJ (2016). Microglial M1/M2 polarization and metabolic states. Br. J. Pharmacol..

[35] Kreiss P (1999). Plasmid DNA size does not affect the physicochemical properties of lipoplexes but modulates gene transfer efficiency. Nucleic Acids Res..

[36] Lukacs GL (2000). Size-dependent DNA mobility in cytoplasm and nucleus. J. Biol. Chem..

[37] McLenachan S, Sarsero JP, Ioannou PA (2007). Flow-cytometric analysis of mouse embryonic stem cell lipofection using small and large DNA constructs. Genomics.

[38] Chen X (2018). Nepetin inhibits IL-1β induced inflammation via NF-κB and MAPKs signaling pathways in ARPE-19 cells. Biomed. Pharmacother..

[39] Hung SSC (2016). AAV-mediated CRISPR/Cas gene editing of retinal cells in vivo. Investig. Ophthalmol. Vis. Sci..

[40] Jo DH (2019). CRISPR-Cas9-mediated therapeutic editing of Rpe65 ameliorates the disease phenotypes in a mouse model of Leber congenital amaurosis. Sci. Adv..

[41] Farjo R, Skaggs J, Quiambao AB, Cooper MJ, Naash MI (2006). Efficient non-viral ocular gene transfer with compacted DNA nanoparticles. PLoS One.

[42] Chalberg TW (2006). Gene transfer to rabbit retina with electron avalanche transfection. Investig. Ophthalmol. Vis. Sci..

[43] Thumann G (2017). Engineering of PEDF-expressing primary pigment epithelial cells by the SB transposon system delivered by pFAR4 plasmids. Mol. Ther. Nucleic Acids.

[44] Hernandez M (2019). Preclinical evaluation of a cell-based gene therapy using the sleeping beauty transposon system in choroidal neovascularization. Mol. Ther. Methods Clin. Dev..

[45] Maeder ML (2019). Development of a gene-editing approach to restore vision loss in Leber congenital amaurosis type 10. Nat. Med..

[46] Ansari AM (2016). Cellular GFP toxicity and immunogenicity: Potential confounders in in vivo cell tracking experiments. Stem Cell Rev. Rep..

[47] Kerur N (2018). cGAS drives non-canonical inflammasome activation in age-related macular degeneration. Nat. Med..

[48] Kumar MV, Nagineni CN, Chin MS, Hooks JJ, Detrick B (2004). Innate immunity in the retina: Toll-like receptor (TLR) signaling in human retinal pigment epithelial cells. J. Neuroimmunol..

[49] Chang YC (2017). High mobility group B1 up-regulates angiogenic and fibrogenic factors in human retinal pigment epithelial ARPE-19 cells. Cell. Signal..

[50] Dib B (2015). Mitochondrial DNA has a pro-inflammatory role in AMD. Biochim. Biophys. Acta Mol. Cell Res..

[51] Jeffries AM, Nitika X, Truman AW, Marriott I (2020). The intracellular DNA sensors cGAS and IFI16 do not mediate effective antiviral immune responses to HSV-1 in human microglial cells. J. Neurovirol..

[52] Bsibsi M, Ravid R, Gveric D, Van Noort JM (2002). Broad expression of Toll-like receptors in the human central nervous system. J. Neuropathol. Exp. Neurol..

[53] Karkhur S (2019). Interleukin-6 inhibition in the management of non-infectious uveitis and beyond. J. Ophthalm. Inflamm. Infect..

[54] Jo DH (2019). Interaction between microglia and retinal pigment epithelial cells determines the integrity of outer blood-retinal barrier in diabetic retinopathy. Glia.

[55] Aksünger A, Or M, Okur H, Hasanreisoğlu B, Akbatur H (1997). Role of interleukin 8 in the pathogenesis of proliferative vitreoretinopathy. Ophthalmologica.

[56] Jonas JB, Tao Y, Neumaier M, Findeisen P (2012). Cytokine concentration in aqueous humour of eyes with exudative age-related macular degeneration. Acta Ophthalmol..

[57] Petrovič GM, Korošec P, Košnik M, Hawlina M (2007). Vitreous levels of interleukin-8 in patients with proliferative diabetic retinopathy. Am. J. Ophthalmol..

[58] Baggiolini M, Walz A, Kunkel SL (1989). Neutrophil-activating peptide-1/interleukin 8, a novel cytokine that activates neutrophils. J. Clin. Investig..

[59] Hess C (2004). IL-8 responsiveness defines a subset of CD8 T cells poised to kill. Blood.

[60] Holtkamp GM, Kijlstra A, Peek R, De Vos AF (2001). Retinal pigment epithelium-immune system interactions: Cytokine production and cytokine-induced changes. Prog. Retin. Eye Res..

[61] Abreu CM (2018). Microglia increase inflammatory responses in iPSC-derived human brainSpheres. Front. Microbiol..

[62] Editas Medicine. Editas Medicine Announces Positive Initial Clinical Data from Ongoing Phase 1/2 BRILLIANCE Clinical Trial of EDIT-101 for LCA10. https://ir.editasmedicine.com/node/10671/pdf (2021).

