# Supplementary material for: Plasmid-mediated gene transfer of Cas9 induces vector-related but not SpCas9-related immune responses in human retinal pigment epithelial cells
Source: Sci Rep. 2022 Aug 1;12:13202. doi: 10.1038/s41598-022-17269-x (PMC9343442; doi:10.1038/s41598-022-17269-x)
Supplement: Supplementary file 1 — Supplementary Information. [file 41598_2022_17269_MOESM1_ESM.pdf]

<u>**Supplemental Figures**</u>

Figure S1

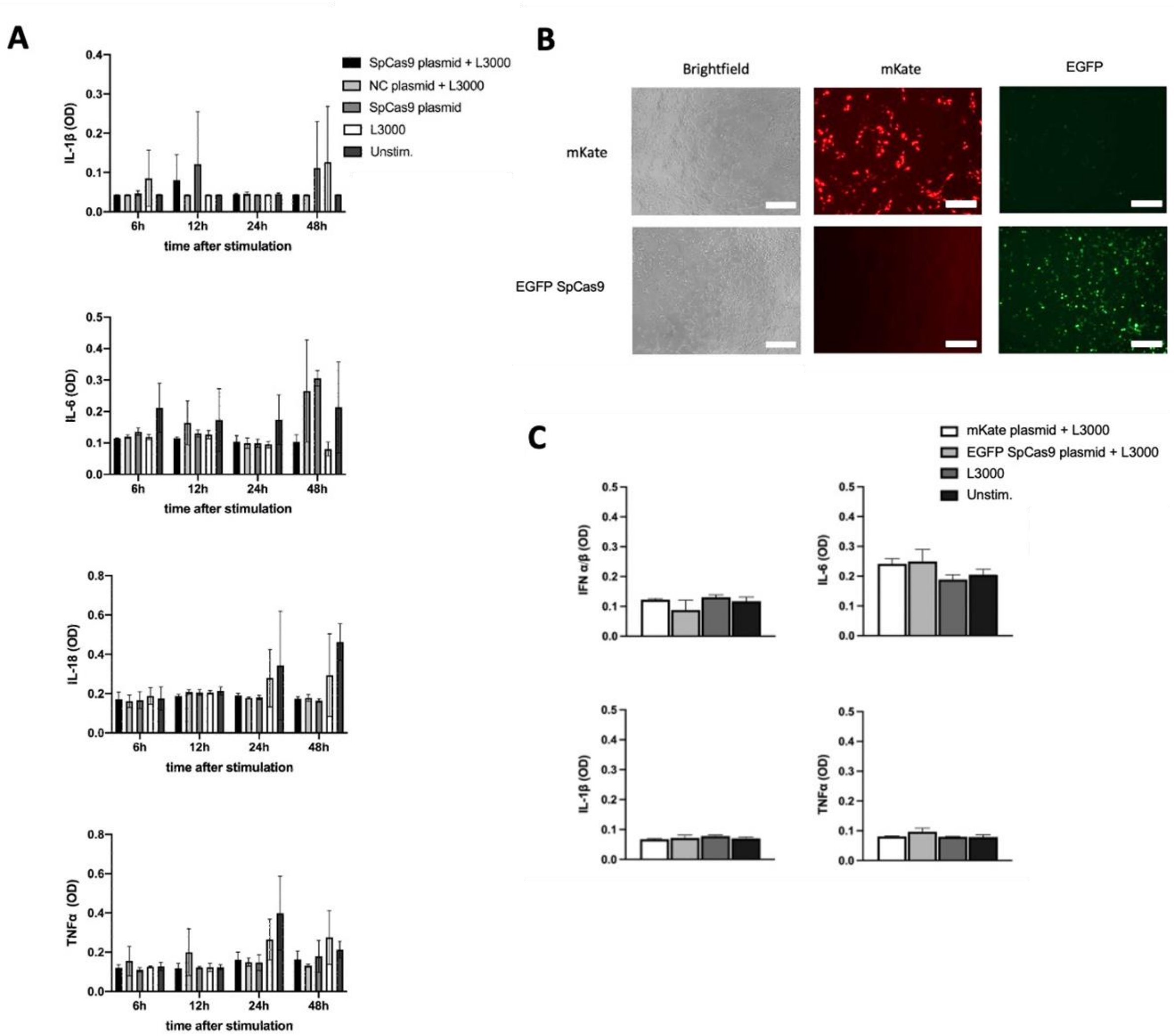

Figure S1: IMhu cytokine production following *Sp*Cas9 plasmid transfection and transfection with additional differently sized plasmids encoding for *Sp*Cas9 and/or fluorescent proteins. (A) IMhu cells were either transfected with *Sp*Cas9 plasmid or NC plasmid, or received L3000 or *Sp*Cas9 plasmid treatment only or were left unstimulated. (B) Microscopic evaluation confirms mKate expression (red) and EGFP expression (green) in IMhu cells 24 hours post-transfection with the mKate plasmid and the EGFP-*Sp*Cas9 plasmid. Nuclei were stained with DAPI (blue). Scale bars: 200 µm. (C) Cytokine release of IMhu cells transfected with mKate plasmid or the EGFP-*Sp*Cas9 plasmid at 24 hours post-stimulation. Cytokines were measured using HEK-Blue IFN α/β, IL-1β, IL-6, IL-18, and TFNα cells. Error bars represent means + SD of n = 3 per group. OD: Optical Density.

Figure S2

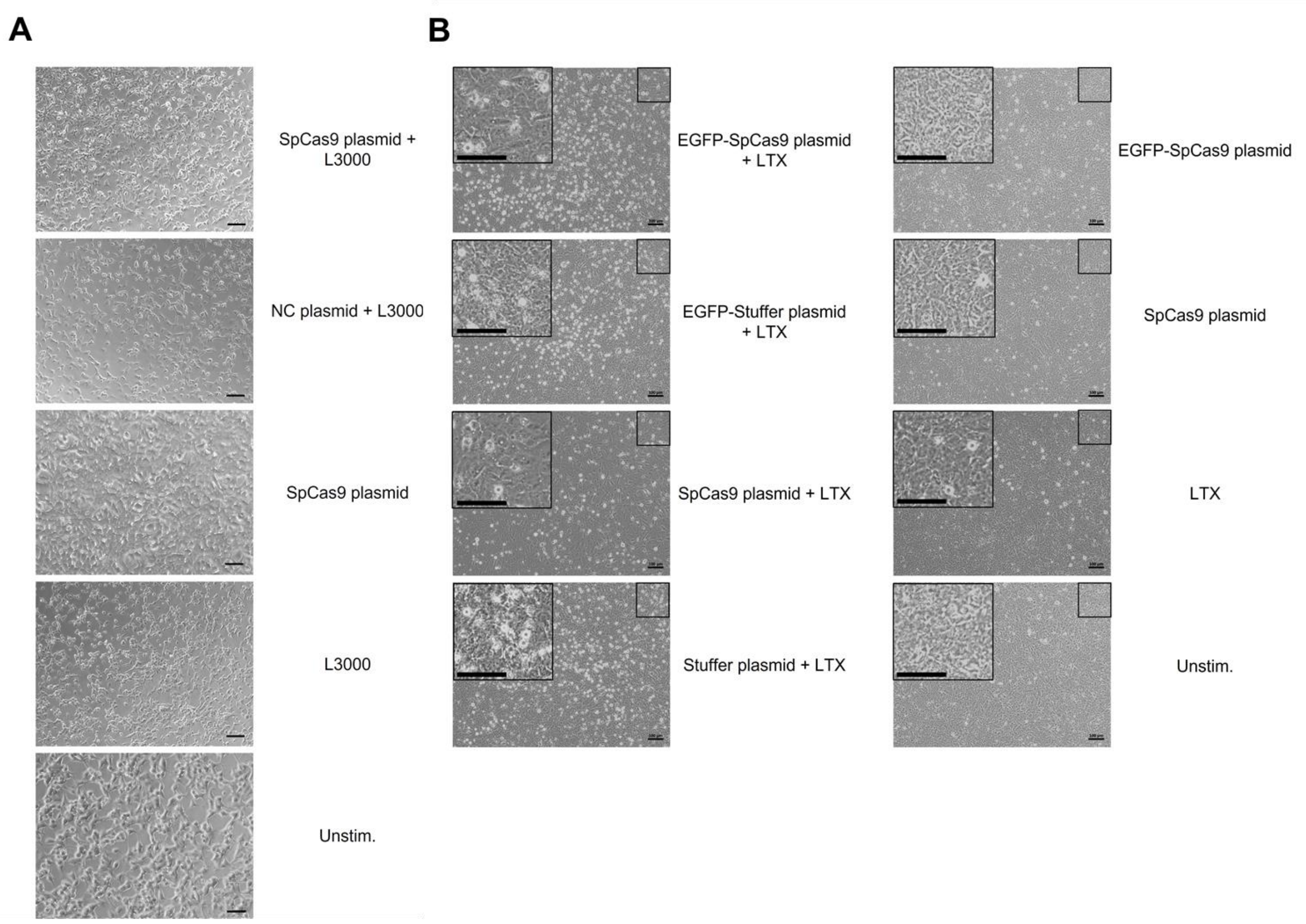

Figure S2: Microscopic evaluation of cell viability of IMhu cells and ARPE-19 cells 24 h after *Sp*Cas9 plasmid transfection. While there is no evidence of cell death in *Sp*Cas9- and NC-plasmid-transfected IMhu cells and the corresponding controls (A), ARPE-19 cultures exhibit spherical but still adherent cells and numerous floating cells in the supernatant which appear to be increased in the *Sp*Cas plasmid- and stuffer plasmid transfected groups (B). Scale bar: 100 µm.
